# Molecular profiling of the developing mouse axial skeleton: a role for Tgfbr2 in the development of the intervertebral disc

**DOI:** 10.1186/1471-213X-10-29

**Published:** 2010-03-09

**Authors:** Philip Sohn, Megan Cox, Dongquan Chen, Rosa Serra

**Affiliations:** 1 Department of Cell Biology, University of Alabama at Birmingham, Birmingham AL, USA; 2 Biostatistics Unit Comprehensive Cancer Center, University of Alabama at Birmingham, Birmingham AL, USA; 3 Biomedical Informatics Program, Clinical Translational Science Institute, West Virginia University, Morgantown WV, USA

## Abstract

**Background:**

Very little is known about how intervertebral disc (IVD) is formed or maintained. Members of the TGF-β superfamily are secreted signaling proteins that regulate many aspects of development including cellular differentiation. We recently showed that deletion of *Tgfbr2 *in Col2a expressing mouse tissue results in alterations in development of IVD annulus fibrosus. The results suggested TGF-β has an important role in regulating development of the axial skeleton, however, the mechanistic basis of TGF-β action in these specialized joints is not known. One of the hurdles to understanding development of IVD is a lack of known markers. To identify genes that are enriched in the developing mouse IVD and to begin to understand the mechanism of TGF-β action in IVD development, we undertook a global analysis of gene expression comparing gene expression profiles in developing mouse vertebrae and IVD. We also compared expression profiles in tissues from wild type and Tgfbr2 mutant mice as well as in sclerotome cultures treated with TGF-β or BMP4.

**Results:**

Lists of IVD and vertebrae enriched genes were generated. Expression patterns for several genes were verified either through in situ hybridization or literature/database searches resulting in a list of genes that can be used as markers of IVD. Cluster analysis using genes listed under the Gene Ontology terms multicellular organism development and pattern specification indicated that mutant IVD more closely resembled vertebrae than wild type IVD. We also generated lists of genes regulated by TGF-β or BMP4 in cultured sclerotome. As expected, treatment with BMP4 resulted in up-regulation of cartilage marker genes including Acan, Sox 5, Sox6, and Sox9. In contrast, treatment with TGF-β1 did not regulate expression of cartilage markers but instead resulted in up-regulation of many IVD markers including Fmod and Adamtsl2.

**Conclusions:**

We propose TGF-β has two functions in IVD development: 1) to prevent chondrocyte differentiation in the presumptive IVD and 2) to promote differentiation of annulus fibrosus from sclerotome. We have identified genes that are enriched in the IVD and regulated by TGF-β that warrant further investigation as regulators of IVD development.

## Background

The vertebral column develops from somites (Reviewed in [1-3]. In response to signals from the notochord and floor plate of the neural tube, the maturing somites will undergo a dorsal-ventral compartmentalization establishing the dermamyotome and sclerotome, the latter forming the future axial skeleton. The ventral part of the sclerotome gives rise to the vertebral bodies and IVD [4]. Due to resegmentation of sclerotome during the formation of the vertebrae, each vertebrae will eventually form from the caudal portion of one somite and the rostral portion of the adjacent somite [5]. The IVD will form at the border of the rostral and caudal domains [6]. IVD are derived from both sclerotome and notochord [7-9]. The outer layer of the IVD, the annulus fibrosus (AF) is derived from sclerotome and provides the structural properties of the IVD. As the vertebral bodies undergo chondrogenesis, notochord cells are removed from the vertebral region and expand into the IVD region to initially form the nucleus pulposus (NP), the central portion of the IVD [10]. TGF-β3 is one of the earliest markers of the developing IVD within the sclerotome [11,12].

Members of the TGF-β superfamily are secreted signaling molecules that regulate many aspects of cell physiology (Reviewed in [13-15]. The family includes three TGF-β isoforms (TGF-β1, 2, and 3), the Activins and Inhibins, Growth and Differentiation Factors (GDFs), and the Bone Morphogenetic Proteins (BMPs). TGF-βs signal through heteromeric serine/threonine kinase receptors. The current model is that TGF-β binds to the TGF-β type II receptor (Tgfbr2) on the cell surface [16]. Tgfbr2 is then able to recruit the type I receptor (Tgfbr1) to form a heterotetrameric complex. Tgfbr2 which is a constitutively active kinase, phosphorylates the type I receptor, activating the type I serine/threonine kinase. Downstream targets of Tgfbr1 then transduce the signal to the nucleus.

All three isoforms of TGF-β are expressed in the developing mouse axial skeleton in distinct and overlapping patterns [11,12,17,18]. Tgfb1 mRNA is localized to intersegmental cells at E12.5 days. By E16.5 days, Tgfb1 mRNA is localized to the ossification centers and perichondrium of vertebrae. At E12.5 days, Tgfb2 mRNA is expressed in all prevertebral segments with the highest levels of expression in the thoracic sclerotome. Tgfb3 mRNA is also expressed in all prevertebral segments marking the location of the future IVD and later becoming restricted to the perichondrium and outer AF of the IVD [11,12]. A systematic study of the expression pattern of the TGF-β receptors during mouse vertebral development has not been reported; however, expression has been detected in the somite and IVD [18-21]. Tgfbr2 is expressed in the adult AF. A decrease in expression is correlated with aging and degeneration of the IVD [22].

The role and necessity of members of the TGF-β superfamily in specific aspects of spinal development and pathology is most clearly illustrated in mice and humans with mutations or targeted deletions in their respective genes [23,24]. Previously, we showed that deletion of Tgfbr2 in Col2a expressing sclerotome resulted in defects in development of the vertebrae and the IVD [25,26]. Specifically, alterations in the AF were detected. The boundary between the IVD and vertebral body was not clearly demarcated and Fibromodulin (Fmod) expression was reduced while expression of Type II collagen splice variant B (Col2; [27]) and staining with peanut agglutinin were increased. The results suggested that TGF-β was required to promote and/or maintain the IVD during development. TGF-β also appears to have a role in maintaining the adult IVD. Polymorphisms within the human Tgfb1 gene have a weak but significant association with Ankylosing Spondylitis and the T29C polymorphism in the Tgfb1 gene is associated with the genetic susceptibility to Spinal Osteophytosis [28,29]. Furthermore, a functional SNP in Cartilage Intermediate Layer Protein (CLIP) is associated with lumbar disc disease. CLIP was shown to co-localize with TGF-β in the IVD and the susceptibility-associated allele showed increased binding to TGF-β and inhibited TGF-β signaling [30].

Very little is known about the molecular mechanisms that govern development of the IVD. One of the barriers to understanding how the IVD develops is a lack of markers to distinguish developing IVD from the cartilage of the vertebrae. In this study, we used laser microdissection and microarray technology to identify genes whose expression was enriched in the developing IVD relative to the adjacent vertebrae. We then used clustering analysis of gene expression profiles in mutant and control vertebrae and IVD to show that in the absence of Tgfbr2, IVD molecularly starts to resemble vertebrae. We then show that treatment of sclerotome with TGF-β results in up-regulation of many IVD enriched genes. Together the data suggest that TGF-β prevents chondrocyte differentiation in the presumptive IVD and promotes differentiation of the AF from sclerotome.

## Results

### Identification of IVD enriched genes

**Figure 1:**
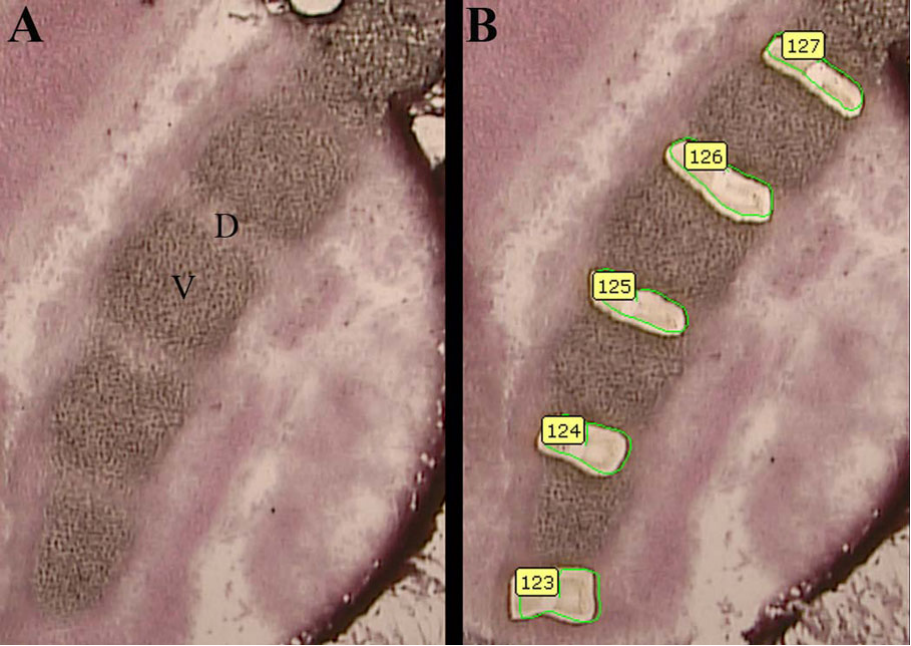
**Laser Microdissection**. Laser Capture Microdissection (LCM) was carried out using a Zeiss/PALM Microbeam Instrument. Lumbar region IVD (D, shown) and vertebrae (V) were microdissected from frozen sections of E13.5 day embryos. (A) shows before and (B) shows after microdissection of IVD. The green circles show the area that was designated for laser cutting.

To identify genes that are enriched in the presumptive IVD relative to the presumptive vertebral body, we used laser capture microdissection followed by microarray analysis. RNA was collected from IVD and vertebrae that were microdissected from the lumbar region of E13.5 day mouse embryos (Figure 1). At this stage, we could just begin to distinguish the presumptive IVD from the adjacent developing cartilage. In addition, the notochord was just beginning to expand into the presumptive IVD region. Microscopically, IVD samples that were collected contained primarily presumptive AF tissue with only a small amount of notochord/NP. Tissue was microdissected from several separate Cre-negative and Cre+Tgfbr2^loxP/loxP ^embryos. After isolation and quality control testing of the RNA we had 3 biological replicates from Cre-negative IVD and vertebrae and Tgfbr2 mutant vertebrae. We had four biological replicates of Tgfbr2 mutant IVD. Each sample was amplified and labelled separately then hybridized to Affymetrix Mouse 430 2.0 GeneChip Arrays (13 arrays total).

**Figure 2:**
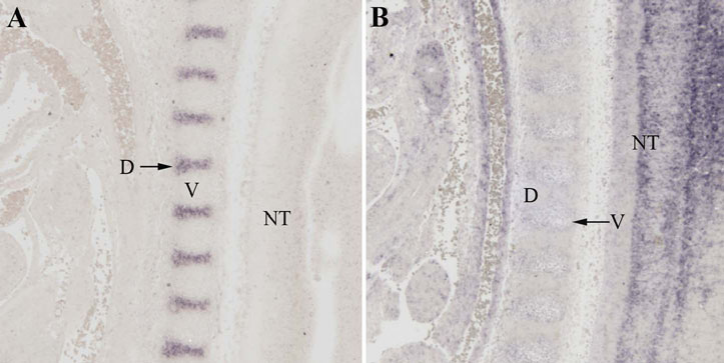
**Verification of localization by in situ hybridization**. (A) A digoxigenin labelled probe to Nfatc1 was hybridized to sections from E12.5 day embryos. Hybridization was visualized as purple staining. Nfatc1 was clearly expressed in the IVD (D, arrow) at this stage. (B) A digoxigenin labelled probe to Ebfa1 was hybridized to sections from E12.5 day embryos. Expression was not detected in the IVD (D) but light purple staining was seen in the vertebral body (V). Staining was also seen in the neural tube (NT) and adjacent blood vessels.

Lists of presumptive IVD and vertebrae-enriched genes were generated by comparing gene expression in wild type (Cre-negative) IVD and vertebrae (Table 1 and additional file 1: supplemental table S1 and additional file 2: supplemental table S2). A total of 263 genes were found enriched in the IVD (additional file 1: supplemental table S1), that is, after normalization and application of statistical cut offs (ANOVA p < 0.05), expression of these genes was 2-fold higher in the IVD than in the vertebrae. One hundred and forty one vertebrae enriched genes were identified (additional file 2: supplemental table S2). Genes whose preferential expression in the IVD was verified either by literature/database search or in situ hybridization (Figure 2) are shown in Table 1. The gene expression databases searched were the Gene Expression Database (GXD) at Mouse Genome Informatics (MGI; http://www.informatics.jax.org) and the Genepaint database (http://www.genepaint.org; [31]. In situ hybridization of Nfatc1, an IVD enriched gene, and Ebf1, a vertebrae enriched gene, are shown in Figure 2. Table 1 thus provides a list of a number of genes that can be used as markers to distinguish developing IVD from vertebrae. The genes include those involved in regulation of the cytoskeleton, extracellular matrix and adhesion, growth factors and regulators of growth factor function, signal transduction, and regulation of transcription. As expected, Tgfb3, was identified in this screen as an IVD enriched gene [11,12].

**Table 1:** IVD enriched, verified genes.

Probe Set ID	Gene Symbol	Fold Difference	Reference
**Cytoskeleton**			

1434326_x_at	Coro2b	3.58	[31]

1456312_x_at	Gsn	2.08	[31]

**Extracellular matrix and adhesion**			

1429214_at	Adamtsl2	2.45	[66]

1448590_at	Col6a1	4.57	[31]

1452250_a_at	Col6a2	3.77	[31]
1426947_x_at		4.11	

1424131_at	Col6a3	4.04	[31]

1427168_a_at	Col14a1	2.84	[67]
1428455_at		2.85	

1436965_at	Emilin3	3.60	[68]

1416164_at	Fbln5	2.79	[69]

1450728_at	Fjx1	2.2	[70]

1415939_at	Fmod	2.72	[42]
1437324_x_at		2.46	
1437685_x_at		2.32	
1437718_x_at		2.35	

1434210_s_at	Lrig1	3.55	[31]
1449893_a_at		4.85	

1421694_a_at	Vcan	2.45	[71]
1427256_at		2.35	
1433043_at		2.09	

**Growth factors and regulators**			

1448421_s_at	Aspn	8.75	[72]

1416652_at		6.04	

1449545_at	Fgf18	2.5	[73]

1419139_at	Gdf5	8.45	[74]

1422053_at	Inhba	4.29	[75]

1417455_at	Tgfb3	2.63	[11]

1425425_a_at	Wif1	7.82	[31]

1448594_at	Wisp1	2.55	[31]
1448593_at		2.81	

**Signal transduction**			

1434034_at	Cerk	2.03	[31]

1448830_at	Dusp1	3.57	[31]

**Transcription factors**			

1433939_at	Aff3	2.20	[76]

1440244_at	Erg	2.91	[58]

1437247_at	Fosl2	6.34	[31]

1434939_at	Foxf1a	2.01	[77]

1418220_at	Foxf2	3.04	[77]

1417621_at	Nfatc1	3.04	Figure 2
1428479_at		4.00	

1449359_at	Pax1	3.04	[4]

1421246_at	Pax9	2.32	[4]

1434286_at	Trps1	3.87	[78]
1438214_at		2.46	

### Effects of losing Tgfbr2 on IVD development

Previously, we generated mice in which Tgfbr2 was deleted using Cre expressed under the control of the *Col2a *promoter. The mice had defects in development of the IVD [25,26]. Specifically, the expression of Fmod, an IVD marker, was reduced and expression of cartilage markers was increased suggesting one of the roles of Tgfbr2 is to prevent chondrocyte differentiation in the AF of the presumptive IVD. Here, we used microarray analysis to compare molecular profiles of normal and mutant IVD to see if we could confirm and extend the previous findings. Two hundred and eleven genes were found to be either up-regulated or down-regulated in the E13.5 presumptive IVD after deletion of Tgfbr2 (additional file 3: supplemental table S3). Several of the IVD markers that were identified in Table 1 and additional file 1: supplemental table S1 were down-regulated in the mutant IVD relative to the controls (Table 2). Likewise, several genes normally enriched in the vertebrae (additional file 2: supplemental table S2) were up-regulated in the Tgfbr2-mutant IVD (Table 3). We confirmed the previous results showing down-regulation of Fmod in the mutant IVD and extended this finding to show down-regulation of other ECM IVD enriched genes including Col6 and Col14. Asporin (Asp), which has been associated with lumbar disc disease in humans [32], was down-regulated in mutant IVD. Trps1, a transcription factor associated with the skeletal defects of Trichorhinophalangeal Syndrome [33], was also down-regulated in mutant IVD.

**Table 2:** Selected IVD enriched genes that are down-regulated in the IVD by deletion of *Tgfbr2*.

Arhgap24	Col14a	Fmod	Hhip	Trps1
Asp	D18Ertd653e	GDF5	Lsamp	Wif1
Col6a1	Erg	Gna14	Ogn	

**Table 3:** Selected vertebrae enriched genes that are up-regulated in *Tgfbr2*-deleted IVD.

Alcam	Dtna	Pcdh17
Asb4	Ebf1	Pik3r1
Bmper	Pcdh9	Prkg2

**Figure 3:**
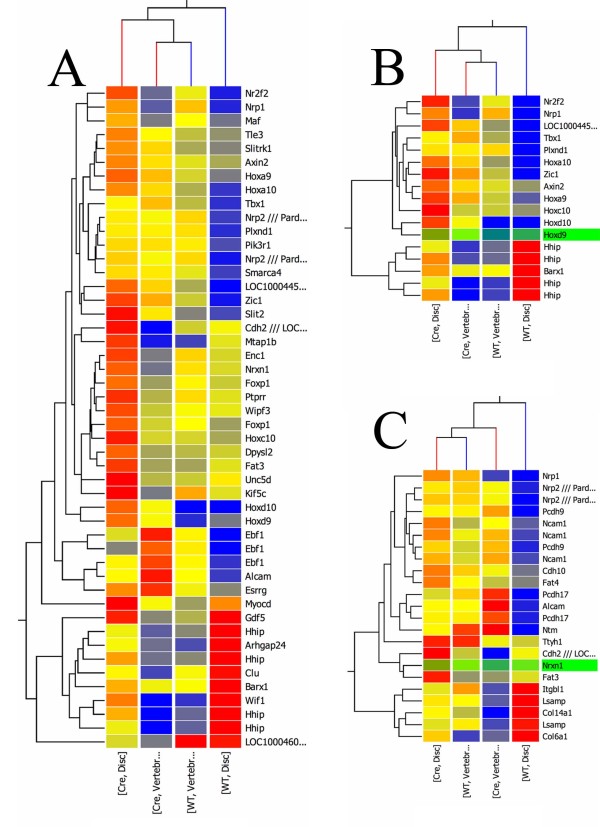
**Hierarchical clustering analysis**. Hierarchical clustering using gene lists containing the GO term multicellular organism development (A), patterning (B), or cell adhesion (C). Red indicates highly expressed genes relative to blue, which represents lower expression. When clustered on the relevant gene lists, mutant IVD (Cre, Disc) clusters with mutant (Cre, Vertebrae) and control (WT, Vertebrae) vertebrae. The condition WT, Disc clusters separately.

A more global analysis of differences in normal and mutant IVD was provided by Gene Ontology (GO) Analysis and hierarchical cluster analysis. First GO analysis indicated that mutations in Tgfbr2 resulted in significant alterations in the IVD in genes associated with development of multicellular organisms, which also included a subset of genes associated with limb development. There are no specific GO terms to define development in the axial skeleton. Genes associated with patterning and cell adhesion were also altered. Hierarchical clustering was used to sort three lists of genes with the GO terms multicellular organism development, pattern specification, and cell adhesion (Figure 3). This analysis clusters conditions that are molecularly most similar together. When genes under the general term of multicellular organism development were used for the cluster analysis, wild type and mutant vertebrae sorted closest together indicating that overall they were molecularly very similar. Mutant IVD clustered closer to the vertebrae samples (Figure 3A). Similar results were obtained for genes under the GO term pattern specification (Figure 3B). In contrast, if the experiment was clustered based on cell adhesion related genes, both IVD and vertebrae were affected so that wild type and mutant IVD did not cluster together and wild type and mutant vertebrae did not cluster together either (Figure 3C). The results support the model in which development and patterning is altered in Tgfbr2-mutant IVD so that it more closely resembles vertebrae than normal IVD.

### TGF-β treated sclerotome preferentially expresses IVD enriched genes

**Figure 4:**
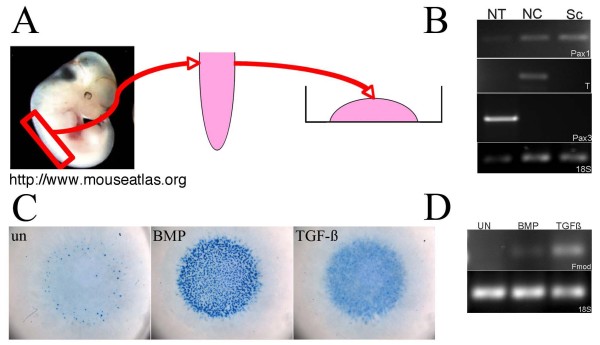
**Sclerotome culture**. (A) Sclerotome was isolated from E11.5 day embyos and grown in micromass culture. (B) RT-PCR to show purity of cultures. RNA was isolated from neural tube (NT), notochord (NC), and sclerotome (Sc) immediately after dissection. cDNA was made from the RNA and expression of Pax1, a marker for sclerotome, Pax3, a marker for myotome and neural tube, and brachyury (T), a marker for the notochord, were determined using PCR. 18S was used as a loading control. Pax1 was expressed in the sclerotome but T and Pax3 were not detected indicating that there was no contamination with myotome, neural tube or notochord in the sclerotome preparation. (C) Alcian blue stained micromass cultures 72 hours after treatment with BMP or TGF-β. Untreated (un) cultures are shown as a control. Very little alcian blue stain was seen in untreated controls. Treatment with BMP resulted in a lot of darkly stained nodules with cartilage morphology. Treatment with TGF-β resulted in an increase in alcian blue stain but discreet nodules with cartilage morphology were not detected. (D) RT-PCR was used to determine expression of Fmod in untreated (UN) and BMP or TGF-β treated cultures. Fmod was induced after treatment with TGF-β.

Next, we wanted to find genes that were potentially directly regulated by TGF-β in sclerotome and determine if TGF-β treated sclerotome acquired characteristics of IVD. To this end, we set up micromass cultures of sclerotome cells dissected from E11.5 day wild type embryos (Figure 4A). This culture system is similar to that commonly used for embryonic limb mesenchyme [34,35]. To determine if there was contamination from other cell layers, for example the myotome, we isolated RNA from the freshly dissected sclerotome as well as the notochord, and neural tube that were left after the dissection. Marker gene expression was used to determine the quality of the dissection (Figure 4B). Pax1 is expressed in sclerotome [4], Pax3 is expressed in the myotome and in the dorsal neural tube [36], and brachyury (T) is expressed in the notochord [37]. In the sclerotome sample, Pax1 was expressed at a high level, as expected. There was little to no expression of Pax3 indicating very little myotome or neural tube contamination in the dissected tissue. The very low level of expression of Pax1 in the notochord and neural tube samples indicates that a small amount of sclerotome was left behind during the dissection.

Cultures were grown in absence of growth factors or in the presence of 50 ng BMP4/ml or 5 ng TGF-β1/ml. After 72 hours in culture, cells were stained with alcian blue (Figure 4C). Cells grown in the absence of growth factors demonstrated a low number of alcian blue stained nodules with similar morphology to the cartilage nodules found in cultures of limb mesenchyme. As expected, treatment with BMP4 resulted in an increase in the number of Alcian blue stained nodules with cartilage morphology [38-40]. Treatment with TGF-β resulted in a different response. Alcian blue staining was present throughout the entire culture but not in discreet nodules. Previously, it was shown that C3H10T1/2 cells, immortalized mesenchymal cells, treated with TGF-β demonstrate a similar response [41]. Furthermore, treatment with TGF-β resulted in increased levels of Fmod mRNA, a previously known marker for IVD (Figure 4D; [42] and Table 1).

Since the TGF-β treated micromass cultures expressed Fmod and did not demonstrate typical cartilage morphology, we next used microarray analysis to test the hypothesis that TGF-β could promote AF differentiation from sclerotome cells. Sclerotome cultures were set up and left untreated or treated with 5 ng TGF-β1/ml or 50 ng BMP4/ml for 8 hours at which time RNA was extracted. The experiment was set up three separate times so that we would have three biological replicates of each condition. After quality control testing of the RNA, each sample was amplified and labelled separately then hybridized to Affymetrix Mouse 430 2.0 GeneChip Arrays (9 arrays total).

**Figure 5:**
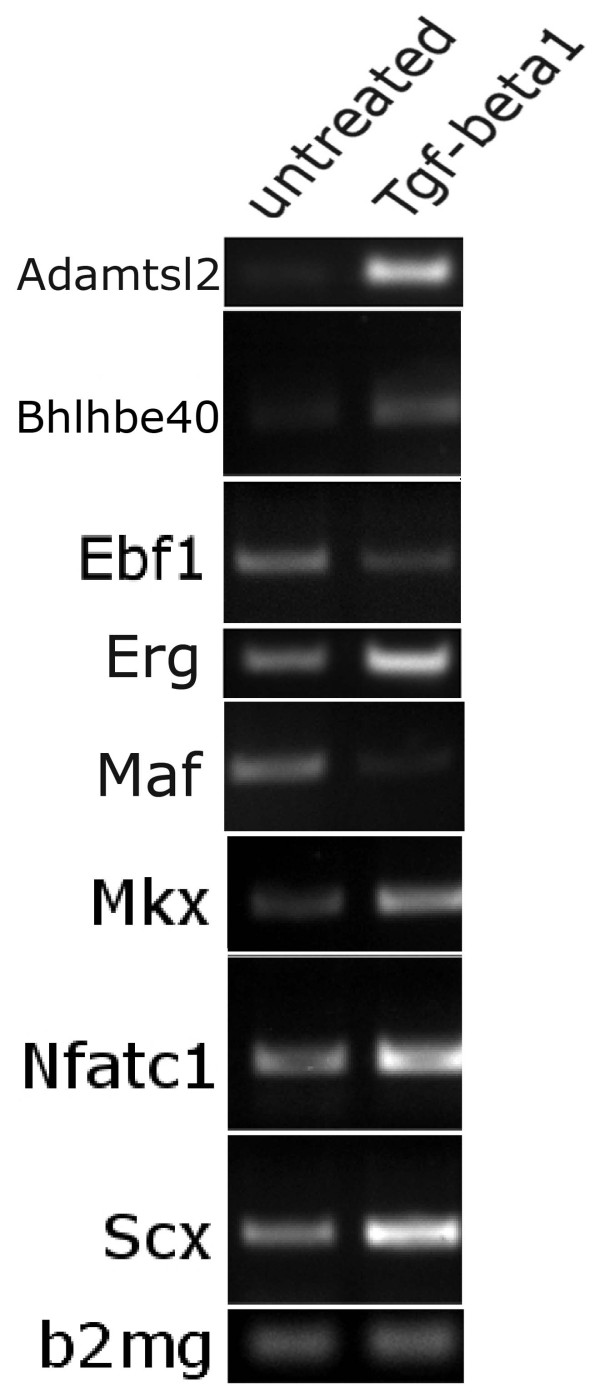
**Verification of regulation of selected genes by TGF-β using RT-PCR**. Sclerotome was treated with TGF-β1 for 8 hours at which time RNA was isolated, cDNA was made and semiquantitative RT-PCR performed. Beta-2-microglobulin (b2 mg) was used as a loading control. Product formation in the linear range is shown.

Gene list for TGF-β regulated and BMP regulated genes were generated (Table 4, 5, additional file 4: supplemental table S4 and additional file 5: supplemental table S5). After normalization and statistical analysis, 281 genes that were either 2 fold up- or down-regulated by TGF-β were identified (additional file 4: supplemental table S4). Four hundred and forty eight BMP regulated genes were identified (additional file 5: supplemental table S5). Many known cartilage markers including Sox5, Sox6, Sox 9, and Aggrecan (Acan), were up-regulated by BMP (Table 5). In contrast, TGF-β did not up-regulate any of these known cartilage markers. Instead, treatment with TGF-β up-regulated many genes that were enriched in the developing IVD, including Fmod and Adamtls2 (Table 4). It is also interesting to note that many vertebrae enriched genes were down-regulated by TGF-β including Maf, a protein known to interact with Sox9 to regulate cartilage gene expression [43]. In addition, at least two tendon markers, Scx and Mkx, were regulated by TGF-β. It was recently shown that TGF-β is also required for normal tendon development [44]. Semiquantitative RT-PCR (Figure 5) and real time PCR (not shown) were used to verify regulation by TGF-β of a subset of genes. As seen in the array data, TGF-β up-regulated Adamtsl2 as well as the transcription factors Bhlhbe40, Erg, Mkx, Nfatc1, and Scx. TGF-β treatment resulted in down regulation of the transcription factors Ebf1 and Maf.

**Table 4:** Selected genes regulated by TGF-β in micromass cultures 2-fold, p < 0.05.

Probe set ID	Gene Symbol	Fold Difference	Direction	Localization	Reference
**Extracellular matrix and adhesion**					

1439827_at	Adamts12	4.38	up	IVD	Table 1

1450627_at	Ank	2.07	up	IVD	[79]

1420569_at	Chad	6.24	up	V (end plate)	[31]

1457296_at	Cilp	3.25	up	IVD	[30]

1416164_at	Fbln5	2.47	up	IVD	Table 1

1422733_at	Fjx1	2.55		IVD	Table 1
1450728_at		2.36	up		

1438966_x_at	Fmod	5.07	up	IVD	Table 1
1456084_x_at		5.01			
1415939_at		5.23			
1437324_x_at		5.77			
1437685_x_at		4.57			
1437718_x_at		5.27			

1434510_at	Papss2	2.02	up	V	[80]

1435603_at	Sned1	6.04	down	V	Table S2

**Growth factors and regulators**					

1449545_at	Fgf18	4.41	up	IVD	Table 1

1421365_at	Fst	2.20	up	IVD	[75]

1438251_x_at	Htra1	3.06	up	IVD	[81]

1421844_at	Il1rap	2.23	up	IVD	Table S1

1448593_at	Wisp1	2.42	up	IVD	Table 1
1448594_at		2.64			

**Signal transduction**					

1423422_at	Asb4	2.14	down	V	Table S2
1433919_at		2.26			

**Transcription factors**					

1418025_at	Bhlhe40	2.42	up	IVD	[82]

1416302_at	Ebf1	2.47	down	V	Table S2
1416301_a_at		2.06			
1448293_at		2.37			

1440244_at	Erg	2.11	up	IVD	Table 1

1456786_at	Ldb2	2.32	down	V	Table S2

1435828_at	Maf	4.72	down	V	[83]
1437473_at		3.05			
1447849_s_at		4.23			
1456060_at		4.26			

1437492_at	Mkx	2.45	up	IVD, Tendon	[84]

1417621_at	Nfatc1	3.2	up	IVD	Table 1
1428479_at		2.71			

1428983_at	Scx	4.03	up	Tendon	[85]

**Unknown function**					

1427182_s_at	D18Ertd653e	3.15	up	IVD	Table S1
1452343_at		2.67			

1441977_at	9630023C09Rik	2.49	down	V	Table S2

IVD = intervertebral disc annulus, V = vertebral cartilage, Table S1 = additional file 1: supplemental table S1, Table S2 = additional file 2: supplemental table S2.

**Table 5:** Selected genes regulated by BMP4 in micromass cultures 2-fold p < 0.05.

Probe set ID	Gene Symbol	Fold Difference	Direction	Localization	Reference
**Cytoskeleton**					

1434326_x_at	Coro2b	2.46	down	IVD	Table 1

**Extracellular matrix and adhesion**					

1449827_at	Acan	3.71	up	V	[71]

1424131_at	Col6a3	3.03	down	IVD	Table 1

1421987_at	Papss2	6.42	up	V	[80]
1434510_at		9.33			
1421989_s_at		7.30			

**Growth factors and regulators**					

1429273_at	Bmper	2.70	up	V	Table S2

1449545_at	Fgf18	6.52	down	IVD	Table 1

1419139_at	Gdf5	2.43	down	IVD	Table 1

1450704_at	Ihh	4.80	up	V	[86]

1428853_at	Ptch1	2.15	up	V (perichondrium)	[31]

**Transcription factors**					

1448601_s_at	Msx1	2.89	up	DM	[87]

1423500_a_at	Sox5	2.35	up	V	[88]
1432189_a_at		2.02			
1455535_at		2.12			

1427677_a_at	Sox6	2.23	up	V	[88]

1447655_x_at		2.17			

1424950_at	Sox9	3.36	up	V	Table S2
1451538_at		2.89			

DM = dorsal trunk mesenchyme, IVD = intervertebral disc annulus, V = vertebrae cartilage, Table S1 = additional file 1: supplemental table S1, Table S2 = additional file 2: supplemental table S2.

**Figure 6:**
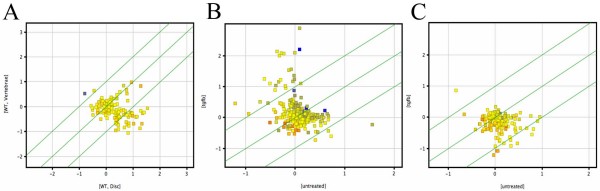
**Scatter plot analysis**. (A) The list of genes that were up-regulated 2-fold by TGF-β was superimposed on the experiment describing differences in wild type vertebrae (WT, vertebrae) and wild type IVD (WT, disc). The dots representing TGF-β genes are preferentially expressed in the IVD. (B) The list of IVD enriched genes was superimposed on the experiment describing genes in cultured sclerotome that were regulated by treatment with TGF-β. The dots representing IVD enriched genes are weighted toward being up-regulated by TGF-β. (C) The list of vertebrae enriched genes was superimposed on the experiments describing genes regulated by TGF-β. Vertebrae enriched genes were more likely to be inhibited by TGF-β.

Scatterplot analysis was used to give a more global analysis of the molecular profile of TGF-β treated sclerotome (Figure 6). First, the list of TGF-β up-regulated genes was superimposed on the experiments describing differential expression of genes in the wild type IVD versus the wild type vertebrae (Figure 6A). In this plot, genes preferentially expressed in the IVD are represented as dots below the center line, with high significance of difference below the outer line. Overall the genes that are up-regulated by TGF-β were skewed toward expression in the IVD supporting the hypothesis that TGF-β can promote IVD phenotype at the molecular level. Next, the list of genes that are preferentially expressed in the IVD was superimposed on the experiment describing genes that are regulated by TGF-β in sclerotome (Figure 6B). In this case, dots above the center line represent genes that are up-regulated by TGF-β and genes below the line are down-regulated by TGF-β. There were more genes in the IVD that were up-regulated than down-regulated by TGF-β. In contrast, if the list of genes that is preferentially expressed in the vertebrae is superimposed on the experiment describing genes that are regulated by TGF-β, genes that are down-regulated by TGF-β predominate. Together the results suggest that TGF-β supports differentiation of IVD from sclerotome.

## Discussion

In this study we used microarray analysis to begin to address the mechanism of TGF-β action in development of the axial skeleton. First, we identified a list of IVD enriched genes that can be used as markers to distinguish developing IVD from the adjacent vertebrae. We also identified a number of genes for which expression is altered in control IVD versus Tgfbr2-deleted IVD. GO analysis indicated that genes associated with development of multicellular organisms, patterning, and adhesion were altered by the loss of Tgfbr2 in the presumptive IVD. Hierarchical clustering analysis indicated that at the molecular level, Tgfbr2 mutant IVD more closely resembled vertebrae than control IVD. The results suggest that Tgfbr2 is required to prevent cartilage formation in the presumptive IVD. We then showed that a number of IVD enriched genes are up-regulated by TGF-β in cultured sclerotome whereas vertebrae enriched genes tended to be down-regulated by TGF-β. The results suggest that TGF-β can also promote differentiation of IVD (AF compartment) from sclerotome.

One outcome of the molecular profiling described here is a list of genes that can be used as markers for developing IVD. It was shown previously that Tgfb3 is one of the earlier markers to denote where the future IVD will form within the sclerotome [11,12]. In addition, we and others have previously used Fmod as a marker for the developing IVD AF [25,42]. Both of these known markers were identified as IVD enriched genes in the screen described here. In addition, the list of markers can be expanded to include GDF5 and Wnt9a, which are also expressed in the interzone of developing synovial joints [45]. Furthermore, several disease-related genes were identified as enriched in the IVD. These include Adamtsl2, Aspn, and Trps1. Adamtsl2 is associated with Geleophysic dysplasia (OMIM: #231050). Patients with Geleophysic dysplasia present with a variety of skeletal abnormalities. It was also recently shown that Adamtsl2 regulates the bioavailabilty of TGF-β resulting in increased TGF-β activity in fibroblasts from Geleophysic dysplasia patients [46]. Polymorphisms in Asp, like Cilp, which is regulated by TGF-β, are associated with Lumbar disc disease (OMIM: #603932). Both Aspn and Clip are extracellular matrix proteins that bind to TGF-β and suppresses its activity [30]. The D14 allele of Aspn is associated with both osteoarthritis and Lumbar disc disease and inhibits TGF-β activity to a greater extent than other alleles [32,47]. Trichorhinophalangeal syndrome, type I (OMIM: #190350) is caused by haploinsuffiency in the transcription factor Trps1. Patients have distinctive craniofacial and skeletal abnormalities. It is not known how Trps1 might regulate development in the axial skeleton [48].

Gene Ontology (GO) analysis of the genes that were regulated in control versus mutant IVD allowed us to determine biological processes that might be altered by loss of Tgfbr2. Eleven GO terms were represented at a significant level in the control versus mutant IVD gene list. The 11 terms could be broadly divided into three categories: multicellular organism development, patterning, and adhesion. Previously we showed that Tgfbr2 is required for normal development of the IVD [25,26]. Using Pax1 and Pax9 as markers of rostral-caudal patterning within the sclerotome we also showed that this patterning was disrupted in Tgfbr2 mutant mice [25]. The list of patterning genes altered by loss of Tgfbr2 is extended in this analysis. We did not previously address alterations in adhesion due to loss of Tgfbr2 in the axial skeleton. The profiling presented here suggests that this would be a logical avenue for future experiments to understand the mechanism of Tgfbr2 action in the development of the axial skeleton and specifically in development of the IVD.

Hierarchical clustering analysis using a list of genes broadly associated with development indicated that by E13.5 days at the molecular level, mutant IVD more closely resembled vertebrae than control IVD. We previously showed that cartilage fills the presumptive IVD space in mutant mice by E14.5 days suggesting that one of the roles of TGF-β in the axial skeleton is to prevent chondrogenic differentiation in the presumptive IVD [25,26]. More recently, we showed that Tgfbr2 also acts to limit chondrogenesis in limb mesenchyme grown in micromass culture [35]. Limb mesenchyme from mice with targeted deletion of Tgfbr2 via Prx1-Cre recombination grown in micromass culture consistently demonstrated an increase in the number of cartilage nodules with increased levels of Alcian blue staining relative to untreated cells from control limbs suggesting TGF-β limits the formation of cartilage from mesenchymal cells. The Prx1Cre; Tgfbr2^lox/lox ^mice also demonstrated a failure to maintain the interzone during development of the joints in the digits. The presumptive interzone was replaced with cartilage resulting in fusion of the synovial joints in the digits. Overall, the data suggest that TGF-β is anti-chondrogenic in limb and sclerotome mesenchyme, allowing development of the synovial and axial joints. The early development of the growth plate of the long bones and vertebral bodies was surprisingly normal in these mice suggesting TGF-β is not required for early development of these structures as previously suspected [25,35]. The effects of TGF-β on mature permanent cartilages, like the articular cartilage, are distinct in that TGF-β appears to maintain the cartilage phenotype and prevent hypertrophic differentiation in these tissues [49-52]. It is known that the response of a cell to TGF-β is dependent on its differentiation status [53].

To identify genes that are potentially involved in the anti-chondrogenic activity of TGF-β, we can compare the lists of vertebrae enriched genes, genes that are up- regulated in the IVD by loss of Tgbr2 in vivo and genes that are directly down- regulated by TGF-β in sclerotome grown in culture. For example, one transcription factor, Ebf1, meets all three of these criteria. Here we showed using in situ hybridization that Ebf1 is expressed at a low level in the presumptive vertebrae as early as E12.5 days. Nothing is known about the role of this factor in development of the axial skeleton; however, it was recently shown that Ebf1 is expressed in cells of the osteoblast lineage and controls osteoblast differentiation [54]. Ebf1-null mice are runted but have an increase in the number of osteoblasts in the bone. We can propose a testable model in which Ebf1 is expressed in the developing vertebrae and is normally down-regulated by TGF-β in the IVD. In the absence of TGF-β, Ebf1 is up-regulated in the IVD region promoting vertebral development. Likewise, another transcription factor, Maf, is down-regulated by TGF-β in cultured sclerotome and up-regulated in mutant IVD relative to control IVD. Maf was previously shown to cooperate with Sox9 to regulate many cartilage-enriched genes [43]. It is possible that down-regulation of Maf by TGF-β is at least partially responsible for its antichondrogenic activity.

Based on results from overlaying lists of genes that are up-regulated by TGF-β with genes that are enriched in the IVD we propose that TGF-β can also promote formation of IVD AF from sclerotome. Previous studies have also shown that adult marrow stromal cells treated with TGF-β more closely resemble IVD than cartilage based on the expression of a smaller set of molecular markers [55]. More recently, it was suggested that TGF-β can either promote cartilage differentiation or shift mesenchymal cell differentiation from a chondrogenic to a fibrous (or tendon) fate depending on the presence of *Tgif1 *and down-regulation of *Sox9 *[56]. Neither expression of Sox9 or Tgif1 were affected by TGF-β in either the cell culture or in vivo experiments performed here suggesting additional modes of regulation exist. We can generate hypotheses about how TGF-β promotes IVD development by comparison of genes that are enriched in the IVD, down-regulated in the mutant IVD, and up-regulated by TGF-β in culture. For example, the transcription factor, Erg, meets all three criteria. It was previously shown that mouse Erg is preferentially expressed in the developing interzone and in the presumptive IVD [57,58]. Over-expression of Erg in mice using the Col2a promoter resulted in a delay in hypertrophic differentiation in the long bones. Furthermore, Tenascin C expression, a marker for articular cartilage, was expanded. It was suggested that the function of Erg was to promote the formation of permanent cartilage. The effects of Erg in the axial skeleton were not addressed but we can propose a testable model in which TGF-β regulates expression of Erg, which in turn could promote formation of the fibrocartilage of the IVD.

## Conclusions

Based on the molecular profiling described here, we propose that TGF-β has two functions in development of the AF in the IVD: 1) to prevent chondrocyte differentiation in the presumptive IVD space and 2) to promote differentiation of AF from sclerotome. We have identified genes that are enriched in the IVD and regulated by TGF-β that warrant further investigation as important regulators of IVD development.

## Methods

### Mouse crosses

All mice in this study were maintained under the guidelines of the Institutional Animal Care and Use Committee of the University of Alabama at Birmingham. Mice in which exon2 of *Tgfbr2 *was flanked with loxP sites (Tgfbr2^f/f^) were obtained from Dr. H.L Moses, Vanderbilt University, Nashville, TN [59]. Tgfbr2^f/f ^mice were mated to transgenic mice that express Cre under the control of the Col2a promoter (obtained from Jackson Labs, ME;[60] to create mice in which *Tgfbr2 *was deleted in sclerotome (Baffi et al 2004, 2006). The genotype of adult transgenic mice was determined by PCR analysis of genomic DNA isolated from tail biopsies as previously described [25,26]. Timed pregnancies were set up by crossing Col2aCre;Tgfbr2^loxP/wt ^mice to Tgfbr2^loxP/loxP ^mice. Noon on the day of the vaginal plug was counted as E 0.5 day. Cre-negative mice were used as controls and sometimes referred to as "wild type". Col2aCre;Tgfbr2^loxP/loxP ^mice were used as the experimental group.

### Laser Microdissection

E13.5 day control and mutant mouse embryos were rinsed in DEPC treated PBS, embedded into OCT and frozen for sectioning. Using a cryostat, 8-12 um sagittal cut frozen sections were collected and placed on PALM PEN-Membrane Slides (P.A.L.M. Microlaser Technologies GmbH, Bernried, Germany). The frozen sections were then quickly dehydrated (70 to 100% EtOH) and stored in Xylene prior to LCM. Laser Capture Microdissection (LCM) was carried out by using a Zeiss/PALM Microbeam Instrument (Microdissection System; Carl Zeiss Microimaging GmbH, Munchen, Germany). The presumptive IVD from the lumbar region (Figure 1) were collected into RNase/DNase free special PALM AdhesiveCaps (P.A.L.M. Microlaser Technologies GmbH, Bernried, Germany). After IVDs were collected, the adjacent presumptive vertebrae were collected into a separate adhesive cap. Collected sample tubes were stored at -80C until RNA was isolated. RNA was isolated using Ambion RNAqueous - Micro Kit (Austin, TX). The optional DNase treatment step was included.

### Sclerotome Micromass Culture

Sclerotome cultures were set up using a method similar to that used for limb micromass cultures [34,35]. Briefly, after removal of the notochord, sclerotome ventral to the neural tube was isolated from E11.5 day mouse embryos. Mesenchymal cells were dissociated into a single cell suspension with incubation in 1 mg/ml collagenase D at 37°C for 30 minutes and reconstituted at a density of 1 × 10^7 ^cells/ml. Twenty microliters of cell suspension was dropped into each well of a 24 well plate. After a pre-incubation time of 1 h at 37°C to allow cells to attach, the cultures were then flooded with F-12:DMEM (3:2) containing 10% FBS, 50 μg/ml ascorbic acid, 10 mM β-glycerolphosphate, 2 mM glutamine, antibiotics with or without 5 ng/ml of TGFβ1 or 50 ng/ml BMP4 (R&D Systems). Cultures were incubated at 37°C in CO_2 _incubator. To stain with Alcian blue, micromass cultures were rinsed with PBS and fixed with 4% paraformaldehyde for 15 minutes at room temperature at which time cells were incubated in Alcian blue staining solution (75%-ethanol Alcian blue solution: 0.1 M HCl = 4:1) at 37°C overnight. Cells were then washed with 70% ethanol and photographed. RNA was extracted from the cells in culture using the standard Trizol method [61]. RNA was Dnase treated and then tested using RT-PCR to assure there was no DNA contamination in the samples.

### Affymetrix Microarrays

The Affymetrix Mouse 430 2.0 GeneChip Array was completed in the Gene Expression Shared Facility located in the Heflin Center for Genomic Sciences at the University of Alabama, Birmingham. The quality of each RNA sample was determined by analysis on the 2100 Agilent Bioanalyzer prior to RNA labeling. Detailed genechip analysis procedures are presented in the Manufacturer's GeneChip Expression Technical Manual (Affymetrix). Briefly, 50 ng of total RNA from each sample was used in a two cycle cDNA amplification protocol using T7-linked oligo dT primers as per the manufacturer's instructions. After the first round of cDNA synthesis an *in vitro *transcription step was utilized to amplify the RNA following which a second round of cDNA synthesis was performed. Subsequently, cRNA was generated and biotin was incorporated into the cRNA strand by standard methods (Affymetrix) followed by cRNA fragmentation, and preparation of hybridization cocktail. The arrays were hybridized overnight at 45°C, and then washed, stained, and scanned the next day. Gene expression levels were extracted using AGCC (Affymetrix GeneChip Command Console).

### Microarray Analysis

Statistical analysis and gene lists for the array experiments were generated using the software package GeneSprings (Agilent, Santa Clara, CA). Bioinformatics analysis including scatterplots, clustering, Gene Ontology (GO) and Gene Set Enrichment Analysis (GSEA), were also performed using GeneSprings (Agilent, Santa Clara, CA). Briefly, to generate gene lists, the raw GeneChip files (.cel) from GeneChip Operating Software (AGCC, Affymetrix, CA) were uploaded to Genesprings, background was subtracted, and data was normalized using the RMA method and default settings in Genesprings. The control or otherwise mentioned group was used as a baseline to calculate the intensity ratio/fold changes of the treated versus the control groups. The ratio was log2-transformed before further statistical analysis. The p-values were obtained by ANOVA assuming unequal variance. Clustering and scatter analysis was also done in Genesprings.

Microarray data was deposited into the Gene Expression Omnibus (GEO; accession number GSE18649).

### In situ hybridization

E12.5 embryos were fixed in 4% paraformaldehyde (PFA) overnight at 4°C then processed for paraffin histology. DIG labeled probes for Nfatc1[62] and Ebf1 [63] were synthesized using T7/Sp6 DIG RNA Labeling Kit (Roche). 5 μm thick tissue sections were dehydrated and fixed for 10 min in 4% PFA then treated with 1 μg/ml proteinase K for 10 min. Sections were post-fixed in 4% PFA for 5 min then treated for 10 min with acetic anhydride in 0.1 M triethanolamine. Pre-treatment was done for 1 hr at 65°C with hybridization buffer (10 mM Tris pH7.5, 600 mM NaCl, 1 mM EDTA, 0.25% SDS, 10% Dextran Sulfate, 1× Denhardt's, 200 μg/ml yeast tRNA, 50% formamide) then incubated overnight at 65°C with probe diluted 1:100 in hybridization buffer. Post-hybridization washes were done in 1× SSC/50% formamide at 65°C for 30 min, TNE (10 mM Tris pH7.5, 500 mM NaCl, 1 mM EDTA) for 10 min at 37°C, TNE/20 μg/ml Rnase A for 30 min at 37°C, TNE for 10 min at 37°C, 2× SSC for 20 min at 65°C, and 2 washes in 0.2× SSC for 20 min each at 65°C. For the antibody incubation, sections were washed in MABT (100 mM Maleic Acid, 150 mM NaCl, 0.1% Tween-20, pH7.5) then incubated for 1 hr in 20% heat inactivated sheep serum (HISS) and 2% blocking solution (Roche) in MABT before adding anti-DIG-AP (1:2500, Roche) in 5% HISS/MABT and incubating overnight at 4°C. Sections were then washed with MABT and placed in BM Purple (Roche).

### RT-PCR

RNA samples were collected from sclerotome micromass cultures untreated or treated with 5 ng/ml Tgfβ1 for 8 hrs, using Trizol (Invitrogen). cDNA was synthesized from equal amounts of total RNA using Superscript III (Invitrogen) with random primers. Semi-quantitative PCR was done using equal amounts of cDNA templates and samples were collected at 25, 30 and 35 cycles. Template amount was normalized using beta2 microglobulin as an internal control. Primer sets used for PCR are listed in additional file 6: supplemental table S6. Primers were designed using NCBI primer BLAST or selected from Primer Bank [64,65].

## Authors' contributions

PS set up the crosses and genotyped all of the mice used for this study, performed laser capture and RNA isolation, set up the sclerotome cultures, treated and isolated RNA from the cultures. MC performed the in situ hybridization experiments and RT-PCR to verify gene expression. DC generated the initial gene lists from the microarray data and assisted with additional statistical and bioinformatics analysis. RS conceived the experimental design, coordinated the experiments, did literature and database searches for gene expression patterns, performed analysis using GeneSprings software, and wrote the manuscript. All authors read and approved the final manuscript.

## Supplementary Material

Additional file 1 **IVD enriched genes**. List of all E13.5 day IVD enriched genes. Click here for file

Additional file 2 **Vertebrae enriched genes**. List of all E13.5 day vertebrae enriched genes. Click here for file

Additional file 3 **Genes differentially expressed in control and *Tgfbr2*-deleted IVD**. List of genes regulated by loss of Tgfbr2 in E13.5 day IVD. Click here for file

Additional file 4 **TGF-β regulated genes**. List of all genes regulated in sclerotome cells after 8 hours of treatment with 5 ng/ml TGF-β1. Click here for file

Additional file 5 **BMP4 regulated genes**. List of all genes regulated in sclerotome cells after 8 hours of treatment with 50 ng/ml BMP4. Click here for file

Additional file 6 **Primers table**. Primers used for PCR. Click here for file
