# Supplementary material for: Molecular profiling of the developing mouse axial skeleton: a role for Tgfbr2 in the development of the intervertebral disc
Source: BMC Dev Biol. 2010 Mar 9;10:29. doi: 10.1186/1471-213X-10-29 (PMC2848151; doi:10.1186/1471-213X-10-29)
Supplement: Additional file 6 — Primers table. Primers used for PCR. [file 1471-213X-10-29-S6.DOC]

| Gene Name | Gene Bank Accession # | Forward Primer (5'-3') | Reverse Primer (5'-3') | product size | Primer Bank ID |
| --- | --- | --- | --- | --- | --- |
| Adamtsl2 | NM_029981.1 | AGACATGCCGCAACCCAGCC | CAGCTCCCACTGCACGGTCC | 226bp |  |
| b2m | NM_009735 | TTCTGGTGCTTGTCTCACTGA | CAGTATGTTCGGCTTCCCATTC | 104bp | 31981890a1 |
| Bhlhbe40 | NM_011498.3 | ACGGAGACCTGTCAGGGATG | GGCAGTTTGTAAGTTTCCTTGC | 103bp | 6755680a1 |
| Ebf1 | NM_007897.2 | GCATCCAACGGAGTGGAAG | GATTTCCGCAGGTTAGAAGGC | 175bp | 6681253a1 |
| Erg | NM_133659.2 | TCACCCCTCAGTCCAAAGCTGC | TCCCCGTTGGTGCCTTCCCA | 217bp |  |
| Fmod | NM_021355.3 | CAACTCCAGCAGCCTTCTTGAG | GCTGATGGAGAACTCATTGATCCTG | 124bp |  |
| Maf | NM_001025577.2 | GCTTCAGAACTGGCAATGAA | GTCTCCACCGGTTCCTTTTT | 113bp |  |
| Mkx | NM_177595.3 | AACCCGTACCCCACCAAGACC | AAGCCCAGCTTAAATCTGGCT | 139bp | 29243954a3 |
| Nfatc1 | NM_198429.1 isoform a | GGAGCGGAGAAACTTTGCG | GTGACACTAGGGGACACATAACT | 94bp | 3643195a1 |
| Scx | NM_198885.2 | CATTTCTCACCTGGGCAATGTGCT | CAACTTTCTCTGGTTGCTGAGGCA | 208bp |  |
